# Factors associated with sexual risk behaviors with non-steady partners and lack of recent HIV testing among German men who have sex with men in steady relationships: results from a cross-sectional internet survey

**DOI:** 10.1186/s12889-015-1987-8

**Published:** 2015-07-24

**Authors:** Sarah C. Kramer, Jochen Drewes, Martin Kruspe, Ulrich Marcus

**Affiliations:** Department for Infectious Disease Epidemiology, HIV/AIDS, STI and Blood-borne Infections Unit, Robert Koch Institute, Berlin, Germany; Public Health: Prevention and Psychological Health Research, Freie Universität Berlin, Berlin, Germany

**Keywords:** HIV, MSM, UAI, Testing, Steady partners, Negotiated safety

## Abstract

**Background:**

Recent evidence suggests that the majority of HIV transmissions among men who have sex with men (MSM) occur between steady partners. We sought to determine factors associated with HIV transmission risks in steady partnerships.

**Methods:**

Data is from the German cross-sectional 2013 Gay Men and AIDS survey. The study population was HIV-negative or untested men reporting a steady partnership and at least one non-steady anal sex partner in the previous year. Bivariate and multivariate logistic regression was used to determine which of several independent variables best predicted both unprotected anal intercourse (UAI) with a non-steady partner and lack of HIV testing in the past year (high-risk outcome group).

**Results:**

The study population consisted of 1731 men. Among individuals in the outcome group (*n* = 271), 67 % reported UAI with a non-steady partner of unknown status and 9 % reported UAI with a non-steady HIV-positive partner in the past 12 months; 55 % considered themselves to be at low risk for HIV acquisition. In multivariate analyses (*n* = 1304), participants were statistically more likely to belong to the outcome group if they reported UAI with their steady partner in the past year (OR = 2.21), did not know their steady partner’s HIV status (OR = 1.98), or agreed that condoms were disruptive during sex (OR = 3.82 (strongly agree), OR = 2.19 (agree)). Participants were less likely to belong to the outcome group if they were out to their primary doctor (OR = 0.54), were well-educated about post-exposure prophylaxis (OR = 0.46), had sought information on HIV in the past year and kept condoms in an accessible place (OR = 0.20), or believed that insisting on condoms would lead partners to assume they were HIV-negative (OR = 0.20). Participants in the outcome group were more likely to say they would use HIV home tests (OR = 1.58) or pre-exposure prophylaxis (OR = 2.11).

**Conclusions:**

Based on our results, we reflect on HIV prevention measures that should be improved in order to better target behaviors that may lead to HIV transmission between MSM in steady relationships. In particular, we highlight the need for multifaceted interventions focusing not only on members of the at-risk community themselves, but on communities as a whole.

**Electronic supplementary material:**

The online version of this article (doi:10.1186/s12889-015-1987-8) contains supplementary material, which is available to authorized users.

## Background

In Germany, as in many developed countries, men who have sex with men (MSM) bear a disproportionate share of the HIV epidemic, with around 75 % of new cases in 2013 estimated to be the result of sexual transmission between men [1]. HIV prevention measures targeted toward MSM often focus on individual-level risks, emphasizing factors such as condom use and overall number of partners, and painting unprotected anal intercourse (UAI) as an inherently risky behavior. However, such efforts may be overly simplistic, and may ignore more complex dynamics occurring between MSM in steady relationships [2, 3]. In light of recent research showing that up to 50–90 % of new infections among MSM may be acquired from steady partners or other partners with whom an individual has multiple sexual encounters [4, 5], particularly those partners thought to be HIV-negative [5], further research on HIV risk among MSM in relationships is indicated.

Research has consistently shown that, all else equal, having multiple sexual partners increases an individual’s risk of acquiring HIV. Both theoretical and observational research suggests that the probability of transmitting HIV to partners is further amplified when these multiple encounters happen over the same period of time [6]. However, many MSM in steady relationships have adopted a range of behavioral strategies such that UAI with a steady partner, even in the presence of anal sex with other, non-steady partners, carries little to no risk for HIV transmission. In 1993, Kippax et al. coined the term “negotiated safety” to describe such agreements [7]. They outline two main conditions that must be met in order for such a strategy to be effective: First, both partners must test negative for HIV and disclose these results to each other. Second, an agreement must be made such that any sex occurring outside of the steady partnership is safe [8]. Such agreements include the decision to engage in sexual activities with only the steady partner, the decision to always use condoms with non-steady partners, and the decision to refrain from engaging in anal sex with non-steady partners. If both of these conditions are met, UAI between two men in a steady relationship becomes a low-risk behavior. However, if either partner does not adhere to these conditions, HIV transmission risk may increase [3, 8]. The European MSM Internet Survey (EMIS) which collected testing data for 38 European countries in 2010 showed that rates of HIV testing in the past 12 months in Germany (33.8 %) fell slightly below the median rate for Europe (34.5 %), and rates of never testing were 30.2 % in Germany compared to an European median of 37.1 %; rates of UAI with non-steady partners were 64.1 % in the German sample compared with an European median of 69.1 % [9]. While all these data are not representative, they show that the situation in Germany is not exceptional for Europe, and that it is important to understand the factors associated with UAI with non-steady partners in the absence of a recent HIV test among MSM in steady relationships if HIV transmission between steady partners is to be reduced.

In this study, we utilize data from the 2013 Schwule Männer und AIDS study (SMA; “Gay Men and AIDS”), a large, cross-sectional internet study of MSM in Germany, to distinguish those participants reporting UAI with at least one non-steady partner as well as no recent HIV test (within the past year), from those participants reporting either only safe sex with non-steady partners or a recent HIV test. We accomplish this goal using bivariate and multivariate logistic regression. Additionally, we present a qualitative analysis of the reasons participants give for having no recent HIV test. Based on our results, we suggest public health interventions that may be used to minimize risks associated with sexual relations outside of existing steady partnerships among MSM.

## Methods

### Data source

Analyses were conducted on data from the 2013 SMA study, an online, cross-sectional study of MSM in Germany funded by the Bundeszentrale für gesundheitliche Aufklärung (BZgA; “Federal Center for Health Education”) [10]. Participants were recruited from various dating sites aimed at gay men and other MSM. After completing the survey, participants could choose to accept a voucher for a free HIV test, redeemable at select testing sites in several larger cities across Germany. Overall, 16,734 men responded to the survey.

The online survey protocol was evaluated and approved by the ethical review board of the Charité University Clinic in Berlin (EA1/266/13). Suggestions by the data protection office of the federal state of Berlin to improve data protection for survey participants were implemented.

### Study sample

We limited our analyses to those participants who reported being in a steady partnership with at least one man. Specifically, participants could indicate being in a monogamous relationship (where no sex with other partners was permitted), being in an open relationship (where sex with other partners was permitted, with or without certain conditions), or having not discussed this with their steady partner. This information was only collected from participants who reported that their HIV-status and the HIV-status of their steady partner were both either negative or unknown. Because the survey asked participants about behaviors within the past 12 months, we also excluded any individuals who did not report being in their current relationship for at least one year. This was done to ensure that any sexual risk or testing behaviors reported did indeed occur while the participant was in a steady relationship, and not before relationship formation. Finally, we considered only those individuals who reported having at least one non-steady anal sex partner in the past 12 months. Although participants adhering to an agreement that does not permit outside partners or only engaging in non-penetrative sexual activities with outside partners are technically also following a negotiated safety regime [8], we were particularly interested in determining those factors associated with safety (condom use and/or testing) among men who engaged in anal sex with non-steady partners. This left us with 1731 participants.

### Dependent variable

We then developed a binary outcome variable. Individuals in the high-risk outcome group reported UAI with at least one non-steady partner, as well as no HIV test, in the past 12 months. These individuals were compared to a reference group consisting of all remaining individuals in the study sample described above. Although many individuals in this reference group do not fulfill both negotiated safety conditions, this approach allowed us to assess those factors specifically associated with the highest level of risk for HIV transmission within steady partnerships.

### Independent variables

Potential predictor variables for our outcome measure included a variety of factors found to be significantly associated with UAI or with lack of HIV testing in previous studies, or else considered to be biologically plausible causal factors for either of these behaviors. We considered: sociodemographic characteristics (age [11], income [11, 12], education [13], size of city of residence, residence in a state in the former Western or Eastern part of Germany, immigration background, sexual orientation); relationship characteristics (type and length of relationship [2, 14, 15], knowledge of partner’s HIV status [15], frequency of UAI with steady partner [13]); psychological and social variables (symptoms or diagnosis of depression or anxiety [15], any professional help for psychological symptoms, internalized homophobia [15, 16], experience of violence due to sexual orientation, “outness” [12], social support [11], HIV-related stigma [17]); use of establishments (such as bars, clubs, or saunas) that cater toward gay men [13]; proportion of sexual partners met on the Internet [18]; knowledge and attitudes toward HIV [19], condom use [14, 15], and antiretroviral therapy (ART) [12, 20]; satisfaction with sex life in general; and substance use [12, 13, 15]. All variables were self-reported. More specifically, “outness” was assessed by asking participants to report the proportion of their family, heterosexual friends, and colleagues who knew their sexual orientation, as well as whether or not their primary doctor was aware that the participant was attracted to men. Participants’ level of social support was assessed by asking participants a) with how many people they felt able to talk about serious personal problems, b) how much interest friends and acquaintances generally show in their lives, and c) how easy it is for them to receive help from family and friends when needed. Responses were reported on a 5-point scale and combined. Internalized homophobia was assessed using an 8-item scale, and HIV-related stigma was assessed using two separate 6-item scales, on which participants could report varying levels of agreement or disagreement. Attitudes toward ART were assessed using a similar methodology. Finally, participant attitudes toward condom use were obtained by asking participants the extent to which they felt condoms disrupted sex, as well as the extent to which they believed partners would make assumptions concerning their HIV status should they insist on condom use. Variables are described in more detail in the Additional file 1.

### Data analysis

We began our analyses by calculating descriptive statistics for our reduced dataset. We then identified candidate variables for our multivariate model by conducting bivariate logistic regression analyses.

For the multivariate regression model, only those variables that were significantly associated (p < 0.05) with the outcome risk behavior in bivariate analyses were considered. Before beginning model selection procedures, any variables with very high rates of missing values were removed from consideration. Relationship type was included in the model from the beginning in order to control for this variable throughout the model-fitting process. Variables were iteratively considered for inclusion or exclusion manually, guided by the add1 and drop1 functions in the R package “stats” [21]. Once all relevant main effects were included, interactions between variables were considered. This process was continued until model fit could not be significantly improved by adding or dropping any variables or interactions. Finally, we observed results for predictors with multiple levels, and combined levels if the direction and magnitude of their association with the outcome variable was similar. The final multivariate model was assessed for fit by observing Pearson and deviance residuals as well as cooks distances. Multicollinearity was explored using variance inflation factors (VIF).

To gain additional insight into novel prevention strategies that may be accepted by and effective among the outcome group, variables found to be significant in bivariate analyses but not judged to be relevant predictors for the main multivariate model were added manually.

We concluded our analyses by assessing the reasons reported by participants for never testing for HIV, or for not testing in the past five years. Similar reasons were combined as described in the Additional file 1. We also observed reasons reported for not accepting the free HIV test voucher at the end of the survey.

## Results

### Descriptive statistics

Participants (*n* = 1731) in our reduced dataset reported a median age of 39; 65.7 % of participants had obtained at least a high school degree, and 57.9 % lived in cities with a population of over 100,000. A small proportion (3.2 %) of respondents were born outside of Germany, and 9.2 % said that one or both of their parents were born outside of Germany. The majority (88.2 %) of individuals in our dataset described themselves as “gay” or “homosexual.” Open relationships were most commonly reported (63.7 %). Overall, just under half (48.4 %) of our study population reported testing for HIV in the past year. The majority (74.5 %) reported engaging in UAI with their steady partner in the past year. Results can be viewed in more detail in Table 1.

**Table 1:** Sociodemographic and other descriptive statistics of the study sample by inclusion in high-risk outcome group

	Outcome Group (*n* = 271)	Reference Group (*n* = 1460)	Total (*n* = 1731)
Relationship type			
Monogamous	60 (22.1 %)	295 (20.2 %)	355 (20.5 %)
Open	147 (54.2 %)	956 (65.7 %)	1103 (63.7 %)
Undecided	64 (23.6 %)	209 (14.3 %)	273 (15.8 %)
Median age (mean)	38 (38.9)	39 (39.3)	39 (39.2)
Size of city of residence			
<100,000	136 (50.2 %)	586 (40.1 %)	722 (41.7 %)
>100,000	132 (48.7 %)	871 (59.7 %)	1003 (57.9 %)
High school degree	155 (57.2 %)	983 (67.3 %)	1138 (65.7 %)
Median monthly income (mean)	2167€ (2445€)	2375€ (2651€)	2375€ (2619€)
Born outside Germany	9 (3.3 %)	47 (3.2 %)	56 (3.2 %)
One or both parents born outside Germany	27 (10.0 %)	132 (9.0 %)	159 (9.2 %)
“Gay” or “homosexual” orientation	238 (87.8 %)	1289 (88.3 %)	1527 (88.2 %)
HIV test in past 12 months	0 (0 %)	838 (57.4 %)	838 (48.4 %)
Any UAI with steady partners (in past 12 months)	230 (84.9 %)	1059 (72.5 %)	1289 (74.5 %)
Any UAI with non-steady partners (in past 12 months)	271 (100 %)	316 (21.6 %)	587 (33.9 %)

Among the individuals in our high-risk outcome group (*n* = 271), 36.5 % reported never testing for HIV (Table 2). Overall, 66.8 % of participants reported at least one instance of UAI with a non-steady partner of unknown HIV status, and 8.9 % reported UAI with an HIV-positive non-steady partner. Despite the prevalence of such risks, 55.4 % of participants in the outcome group believed themselves to have been at low risk for HIV in the past 12 months.

**Table 2:** Behavioral characteristics of the outcome group (*n* = 271) in the 12 months before completing the survey

Never tested	99 (36.5 %)
Any UAI with steady partner	230 (84.9 %)
UAI with >1 non-steady partner	151 (55.7 %)
UAI with >5 non-steady partners	46 (17.0 %)
Any UAI with partner of unknown status	181 (66.8 %)
Any UAI with HIV-positive partner	24 (8.9 %)
Perceived risk in past 12 months	
Low	150 (55.4 %)
Medium	83 (30.6 %)
High	15 (5.5 %)

### Factors associated with belonging to outcome group

In bivariate analyses (Table 3), the odds of a participant belonging to the outcome group were significantly higher among participants who: had not discussed relationship type with their steady partner; reported any UAI with their steady partner; reported being unaware of their steady partner’s HIV status or that their steady partner had never been tested; lived in a city with fewer than 100,000 inhabitants; had not graduated from high school; were “out” to less than half of their heterosexual friends; reported lower levels of social support; reported higher internalized homophobia; had a primary care doctor who did not know the participant was attracted to men; did not keep condoms in an easily accessible location; strongly or partially felt that condoms disrupt sex, or else had no clear opinion; felt that sexual partners would assume he was HIV-positive, or would not automatically assume he was HIV-negative, if he insisted on using condoms; did not feel well-informed about ART; did not feel well-informed about ART’s ability to reduce HIV infectivity; felt less concerned about HIV in general due to ART availability; had not sought out information about HIV in the past year; or were not aware of post-exposure prophylaxis (PEP).

**Table 3:** Results of bivariate and multivariate analyses for inclusion in outcome group. Variables in bold remain significant in the multivariate model.

	Unadjusted OR (95 % CI)	Adjusted OR (95 % CI)
Relationship type		
Monogamous	1.00 (ref)	1.00 (ref)
Open	0.76 (0.55-1.05)	0.87 (0.57-1.36)
Undecided	1.51 (1.02-2.24)	1.29 (0.77-2.19)
City of residence >100,000 people	0.65 (0.50-0.85)	
High school diploma	0.65 (0.50-0.85)	
**Any UAI with steady partner in past 12 months**	2.13 (1.51-3.07)	2.21 (1.42-3.56)
Steady partner HIV status		
Negative	1.00 (ref)	1.00 (ref)
Never tested	2.00 (1.39-2.84)	1.57 (0.96-2.54)
**Unknown**	2.77 (1.94-3.92)	1.98 (1.21-3.19)
Less than half of heterosexual friends are aware of participant’s sexual orientation	1.52 (1.06-2.15)	
**Doctor knows participant is attracted to men**	0.51 (0.39-0.66)	0.54 (0.38-0.76)
Social support		
High	0.47 (0.30-0.75)	
Medium	0.73 (0.48-1.14)	
Low	1.00 (ref)	
Internalized homophobia	1.17 (1.04-1.31)	
Condoms in house or bag	0.30 (0.20-0.46)	1.44 (0.61-3.58)
**Feels that condoms disrupt sex**		
Strongly agrees	5.18 (3.53-7.64)	3.82 (2.36-6.24)
Partially agrees	2.13 (1.51-3.04)	2.19 (1.44-3.35)
Does not agree	1.00 (ref)	1.00 (ref)
Difficult to answer	2.10 (1.05-3.94)	2.45 (1.12-5.06)
Feels partners will assume he is HIV-positive if he insists on condoms	2.63 (1.79-3.82)	
**Feels partners will assume he is HIV-negative if he insists on condoms**	0.67 (0.51-0.88)	0.20 (0.08-0.48)
Has ever heard of ART		
Yes, feels well-informed	0.37 (0.19-0.75)	
Yes, but does not feel well-informed	0.66 (0.35-1.31)	
No	1.00 (ref)	
Has heard that ART can reduce infectivity		
Yes, feels well-informed	0.54 (0.38-0.77)	
Yes, but does not feel well-informed	0.75 (0.54-1.05)	
No	1.00 (ref)	
Reduced concerns about HIV due to ART availability	1.62 (1.30-2.01)	1.22 (0.86-1.70)
Sought information on HIV in past year		
Regularly	0.26 (0.14-0.47)	2.12 (0.77-6.02)
Occasionally	0.40 (0.29-0.55)	
Never	1.00 (ref)	1.00 (ref)
Has ever heard of PEP		
**Yes, feels well-informed**	0.43 (0.28-0.63)	0.46 (0.28-0.75)
Yes, but does not feel well-informed	0.66 (0.48-0.90)	0.77 (0.53-1.12)
No	1.00 (ref)	1.00 (ref)
Feels partner will assume he is HIV-negative if he insists on condoms * Reduced concern about HIV due to ART availability (interaction)	NA	2.17 (1.30-3.66)
Sought any information on HIV in past year * Condoms in house or bag (interaction)	NA	0.20 (0.07-0.58)

* indicates an interaction

In multivariate analysis (Table 3), participants were more likely to belong to the outcome group if they reported any UAI with their steady partner, reported their partner’s HIV status to be “unknown,” or either felt that condoms disrupted sex or had no opinion. Odds of being in the outcome group were reduced among those whose primary doctor knew that they were attracted to men, who believed that a partner would assume they were HIV-negative if they insisted on a condom, who felt well-informed about PEP, and who actively sought out information about HIV in the past year and also kept condoms in their house or bag. Among those who believed a partner would assume he was HIV-negative if he insisted on using condoms, reduced concern about HIV due to ART increased the likelihood of belonging to the outcome group. Due to missing values, only 1304 participants were included in the final model. Overall, our model fits the data fairly well (chi-square goodness of fit: 1354.328, *p* > 0.05) and has high predictive value (leave-one-out cross-validation prediction error: 0.109). All VIFs are below 2, indicating a lack of multicollinearity.

### Home testing and PrEP

In bivariate analyses, we found that individuals in the outcome group were significantly more willing to use home tests for HIV (OR = 2.00 (1.41–2.88)) and pre-exposure prophylaxis (PrEP; OR = 2.74 (1.82–4.17)) than those in the reference group. After controlling for variables included in our multivariate model, these associations remained significant (home tests: OR = 1.58 (1.01–2.53); PrEP: OR = 2.11 (1.29–3.47)).

### Reasons for not testing

**Fig. 1:**
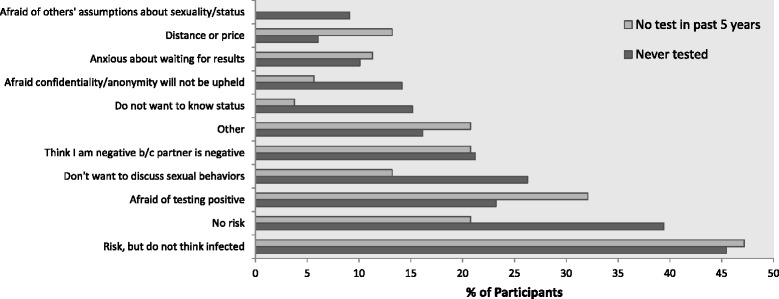
Reasons for never testing and not testing in past 5 years. This figure displays the reasons endorsed by 99 participants who indicated that they had never been tested for HIV, as well as those reasons selected by 53 participants who indicated that their last HIV test occurred over 5 years before beginning the survey. Participants were allowed to select multiple responses

Among the 271 individuals in our outcome group, 99 reported never testing and 53 reported that their last test was more than 5 years ago. Among both groups, the most frequently reported reason for never testing was not thinking one was infected, despite previous risk behavior. Another common reason reported was that participants did not believe they were at risk of contracting HIV. Many individuals also reported that they believed themselves to be HIV-negative because their steady partner was, despite having UAI with non-steady partners, or that they were afraid of testing positive. Individuals who had never tested in particular reported not wanting to discuss their sexual behaviors (Fig. 1).

**Fig. 2:**
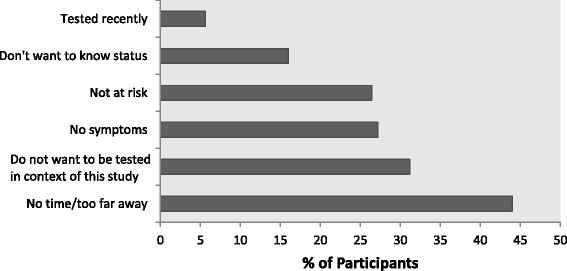
Reasons for not accepting voucher for free HIV test. This figure displays the reasons endorsed by 125 out of 190 participants who did not accept the voucher for a free HIV test offered to participants at the end of the survey. Participants were allowed to select multiple responses

Of 190 participants in the outcome group who did not accept the free HIV test voucher, 125 provided a reason (Fig. 2). The most commonly reported reason was that the participant did not have time or that testing facilities were too far away (*n* = 55). Despite the fact that individuals in this outcome group by our definition have recent high-risk behavior and have not been recently tested, 33 participants reported not accepting the voucher because they did not believe themselves to be at risk, and 7 said that they had already been tested recently.

## Discussion

Our multivariate analysis revealed a number of factors associated with UAI with non-steady partners and no recent HIV test among MSM in a steady relationship with another man. Importantly, we found that men in our high-risk outcome group were significantly more likely to report UAI with their steady partner during the past year, and therefore theoretically more likely to transmit any newly acquired STIs, including HIV, to their steady partner. This further highlights the importance of strengthening risk-reduction strategies that target individuals in the outcome group.

After controlling for the other variables included in our model, we found that relationship type was no longer significantly associated with outcome group inclusion. Thus, our results should help challenge the belief of some researchers that any non-monogamous relationship is inherently risky, or, similarly, that monogamous relationships are inherently safe [2, 3, 8].

The other variables included in our final multivariate model point to a wide range of potential interventions that may reduce risks associated with concurrent sexual relations with both steady and non-steady partners and increase HIV testing. For example, our finding that participants who felt that condoms disrupt sex were more likely to report UAI with non-steady partners is neither new nor surprising [14, 15]. However, it does emphasize the importance of public health initiatives that aim to change attitudes toward condoms. For example, condom use with non-steady partners may be promoted as a selfless act, undertaken by HIV-negative men in relationships to protect not only themselves, but their steady partner [22, 23]. Furthermore, public health organizations can promote options, such as negotiated safety, which do not rely on always using condoms. Since these strategies allow individuals to forgo condoms with their steady partner, they may be particularly appealing to MSM with negative attitudes toward condoms. In this case, campaigns should include effective guidance on negotiating safer sex with non-steady partners, as well as on disclosing and handling breaks in the agreement with the steady partner. Ideally, relevant skills would be promoted among single MSM as well as MSM in relationships, so that individuals can negotiate appropriate agreements early in new relationships, before initiating unprotected sexual activity [14].

Additionally, we found that men reporting that their partner’s HIV status was “unknown” were significantly more likely to belong to the outcome group, suggesting a lack of communication between partners. Closer inspection revealed that this relationship existed only among participants who also did not know their own status. Thus, a primary reason for lack of communication about HIV status between MSM and their steady partners may be reluctance to admit that they themselves are unaware of their status. To encourage communication, HIV prevention campaigns should promote HIV status awareness, as well as mutual status disclosure or testing together when starting a new relationship.

We also found that the risk of belonging to the outcome group was reduced when the participant reported that his primary doctor knew he was gay. We expect that this association is due to two main factors. First, individuals who share this information with their doctors may tend to be more “out” about their sexuality than those who don’t. Additionally, a doctor who knows that his or her patient has sex with other men may be more likely to discuss HIV prevention strategies and HIV testing. In order to encourage MSM to disclose their sexuality to their doctors, it seems particularly important that stigma, both against HIV and homo- and bi-sexuality, be addressed among both medical professionals and the general public. Additionally, it is crucial that physicians feel comfortable taking sexual histories and are trained to provide necessary guidance should risk behaviors come to light.

The need to combat HIV-related stigma may also be indicated by the significant, negative association between outcome group status and believing that partners will assume the participant is HIV-negative if he insists on using condoms. Individuals who perceive that a sexual partner may assume that they are HIV-positive if they insist on using a condom may be more likely to refrain from pushing for condom use due to fear of negative backlash from the partner [24]. Expressing reduced concerns about HIV due to the availability of ART, however, eventually cancels this effect out. This is likely because individuals with more faith in ART are less concerned about engaging in risky sexual behaviors [20]. Thus, so-called “treatment optimism” must also be addressed by future public health campaigns.

We also found that participants who felt well-informed about PEP were less likely to belong to the outcome group. This may indicate that individuals who are well-informed about HIV prevention and treatment methods in general are also those individuals who tend to be tested for HIV and to take precautions during sex with non-steady partners. Thus, we find no evidence that disseminating information on the preventative potential of ART in and of itself has a negative impact on sexual risk behaviors or HIV-testing. Education on the entire range of preventive options to reduce the risk of HIV acquisition should continue to be an integral part of public health strategies. The need for more education, particularly concerning HIV risks, is again highlighted by our descriptive statistics, which reveal that, despite significant rates of UAI with partners of unknown or positive status, 55.4 % of our outcome group considered themselves to be at low risk of contracting HIV in the past year.

However, our results suggest that even individuals who actively seek out information about HIV are not statistically less likely to belong to the outcome group. Our finding that seeking information on HIV and having condoms in one’s house or bag only significantly reduced the risk of belonging to the outcome group when reported together suggests that freely available condoms may only be helpful to those who have recently sought out information about HIV, and simultaneously that HIV education may only reduce HIV risk behaviors if condoms are available should a sexual situation arise. Public health agencies should ensure that educational campaigns include easy access to affordable condoms, and vice versa. From a broader perspective, these results suggest that educational campaigns concerning any HIV prevention strategy be accompanied by resources that increase the ease of adhering to the strategy in question.

After controlling for the above mentioned variables, individuals in the outcome group remained more willing to use HIV home tests and PrEP. This suggests that, although individuals in this study tend to have negative attitudes toward condom use and to be reluctant to use currently available HIV testing options, there are other promising HIV prevention and testing strategies that may be successfully implemented among this group. However, it is important to note current problems with HIV home tests: the tests are expensive, do not detect early HIV infection, and individuals using home tests will not have access to immediate professional risk and emotional counseling after receiving their results [25]. Similarly, the long-term efficacy of PrEP is unknown; additionally, it is unaffordable for many individuals at its current price levels, may require pills to be taken according to a strict daily schedule, and may cause side effects [26, 27]. It is therefore crucial that such strategies continue to be improved upon to increase both their effectiveness and their availability to at-risk individuals and their partners.

Reasons reported by participants in the outcome group for never being tested for HIV, or for not being tested in the past five years, were remarkably similar. For both testing behaviors, reporting a perceived lack of risk, believing oneself to be HIV-negative despite acknowledged risk behavior, and believing oneself to be HIV-negative because a steady partner is HIV-negative were among the most commonly endorsed reasons, again emphasizing the need to educate the community more fully on what behaviors carry a risk of HIV transmission, and to encourage individuals to test after engaging in these behaviors, whether or not they believe themselves to be infected. Additionally, MSM in steady relationships should be encouraged to communicate with their partners to determine how recent a partner’s HIV-negative test was, and whether any of their partners had UAI with anyone else since their last test. Another reason commonly chosen was fear of testing positive. Such fears could possibly be alleviated by emphasizing the numerous therapeutic options available for individuals aware of their HIV-positive status. Additional emotional support for those undergoing testing may also be indicated; among MSM with steady partners, this could be accomplished by encouraging partners to test together [28]. Finally, participants commonly reported not wanting to talk about or be lectured about their sexual behaviors. Once again, our results emphasize the importance of respectful and non-judgmental attitudes among health professionals.

Among those who did not accept the voucher for a free HIV test, the most commonly cited reason was that they had no time to be tested, or that testing facilities were too far away. These results indicate the importance of improving availability and efficiency of HIV testing, although it is important to note here that the vouchers could only be used at certain testing sites, and that these responses therefore do not necessarily indicate that testing opportunities in Germany are lacking in general. Many participants also reported that they did not want to be tested due to a lack of symptoms, indicating a need to clear up misconceptions concerning how HIV presents itself. Finally, it is interesting to note that several individuals reported that they were either not at any risk or that they had been tested recently; these results point again to the need for more education on HIV transmission risks and clearer recommendations for testing.

### Limitations

It is important to acknowledge that, as this survey was cross-sectional, it is impossible to draw any causal conclusions from our results. Additionally, the survey did not collect information on relationship-level characteristics such as trust, satisfaction, or commitment, all of which may impact risk and testing behavior [29], and we have no data on the sexual behaviors of participants’ steady partners. We also emphasize that our classification of participants into the high-risk outcome group may not have been completely accurate. For example, some individuals in the outcome group reported no UAI with their steady partner, and are therefore at very low risk of transmitting HIV to this partner, regardless of behavior with non-steady partners (see Additional file 1). Furthermore, we note that our reference group includes not only individuals who fully adhered to a negotiated safety strategy (reported a recent HIV test and no UAI with non-steady partners), but also individuals who adhered to only one of these tenants. For this reason, some individuals placed into our lower-risk reference group may not necessarily have negligible risk for HIV acquisition. By including only those individuals reporting steady relationships lasting at least a year, we fail to consider individuals in newly-formed relationships, who may be more likely to acquire HIV from or transmit HIV to a potentially discordant steady partner [30], and who may therefore represent an important subset of HIV transmissions between steady partners. Additionally, it is important to realize that condoms are susceptible to breakage and slippage, which may increase risk of HIV transmission [31]. We also note that, as this survey recruited participants from the Internet, our results may not be generalizable to the MSM population as a whole. Finally, as our dependent variables were all self-reported, it is likely that some degree of recall and social desirability bias is present.

## Conclusions

Our results indicate that, if HIV transmission is to be reduced and testing increased among MSM in steady relationships in Germany, a multifaceted approach including a variety of public health strategies will likely be required [13, 32, 33]. Furthermore, it is important that certain measures, particularly those combating stigma, are targeted not only toward MSM, but to the population in general. Healthcare workers and other employees at testing facilities in particular should be educated on how to recognize HIV risk and discuss prevention and testing measures in a clear, understanding, and non-judgmental way.

In addition to the improvement of public health measures, future research should more closely analyze both individual- and partnership-level factors. Longitudinal studies are also needed in order to clarify the direction of associations found here. Finally, the development of negotiated safety strategies by the MSM community should serve to remind us of the need to communicate meaningfully with members of the at-risk population. This will allow us not only to learn of novel prevention ideas not thought of by the research community, but also to better understand which strategies are most likely to be accepted and effective among MSM.

## Additional file

Additional file 1:
**OnlineSupplement.pdf describes the variables considered in this study in more detail, and provides results for a sensitivity analysis in which all individuals reporting no UAI with their steady partner in the past year were removed from the analysis.**
