# Supplementary material for: Factors associated with sexual risk behaviors with non-steady partners and lack of recent HIV testing among German men who have sex with men in steady relationships: results from a cross-sectional internet survey
Source: BMC Public Health. 2015 Jul 24;15:702. doi: 10.1186/s12889-015-1987-8 (PMC4513622; doi:10.1186/s12889-015-1987-8)
Supplement: Additional file 1: — OnlineSupplement.pdf describes the variables considered in this study in more detail, and provides results for a sensitivity analysis in which all individuals reporting no UAI with their steady partner in the past year were removed from the analysis. [file 12889_2015_1987_MOESM1_ESM.pdf]

SUPPLEMENTARY MATERIAL

1. Independent Variables

Sexual risk and HIV testing behaviors

Participants reported the number of non-steady partners with whom they had anal sex and UAI in the past 12 months. Additionally, participants were asked whether, in the past year, they had UAI with someone whose HIV test result they did not know, or with anyone they knew to be HIV-positive.

Participants indicated, on a scale of 0 (“no risk”) to 10 (“very high risk”), how high they believed their risk of acquiring HIV in the past year to be. We categorized responses of 0-2 at “low perceived risk,” 3-6 as “medium perceived risk,” and 7-10 as “high perceived risk.”

Similarly, participants were asked whether they had ever been tested for HIV and, if so, when their most recent test occurred. Based on this information, participants were categorized as having had a recent HIV test (past 12 months), as having previously been tested but having no recent test, or as having never tested for HIV. Participants also reported whether or not they received their result at their last test.

Sociodemographic variables

Demographic variables were directly reported by participants.

Relationship characteristics

Similarly, participants were asked to indicate how long they had been in their current relationship with another man, and whether the relationship was monogamous (no sex with other men), open (sex with other men permitted), or had not been discussed with the steady partner. Participants self-reported frequency of UAI with their steady male partners in the past year (1-2 times, 3-4 times, 5-10 times, every month, or every week). Participants were also asked if they were aware of their steady partner’s HIV status, and could report:

1. He has never been tested for HIV.
2. Yes, his last test was HIV-negative.
3. No, but I think he is HIV-negative.
4. No, I have no interest in knowing his status.
5. No.

For analyses, the last three options were combined as “unknown.”

Psychological and social measures

Psychological symptoms were assessed by first asking participants to rate how often during the past two weeks they experienced the following symptoms of depression and anxiety:

1. Reduced interest or happiness with daily life
2. Feelings of defeat, sadness, or hopelessness
3. Feeling nervous, afraid, or tense
4. Feeling unable to control worries

Responses were coded and added. Participants were then asked to indicate whether they had ever accessed any sort of professional help for psychological symptoms, and if they had ever been diagnosed with a mental illness.

"Outness" was assessed in four items, each included separately in our analyses. The first three items asked participants what proportion of their family, heterosexual friends, and colleagues knew their sexual orientation. Participants were also asked whether or not their primary doctor knew that they were attracted to men; this variable was coded as "yes" if the participant reported that he had spoken about his sexual orientation with his doctor, or if he had not but believed his doctor knew, and as "no" otherwise ("no," "I don't know," "no primary care doctor").

Participants' social support was assessed based on the following three questions, with responses on a five point scale:

1. How many people are you close enough with, that you can talk to them about serious personal problems?
2. How much interest do friends and acquaintances usually show in your life?
3. How easy is it for you to receive practical help from friends and acquaintances when you need it?

Responses to the last two questions were recoded and responses were added. Individuals were classified as having weak (1-8), moderate (9-11), or strong (12+) social support.

Gay- and HIV-related stigma

To assess internalized homophobia, participants were asked to rate, on a scale of 1 to 7, the extent to which they agreed with the following items:

1. I feel uncomfortable in the presence of "feminine" gay men.
2. I feel comfortable in gay pubs and bars.
3. I feel uncomfortable in the presence of openly gay men.
4. Being seen in public with an obviously gay person does not bother me.
5. Speaking publicly about homosexuality does not bother me.
6. I feel comfortable as a homosexual man.
7. I consider homosexuality to be morally acceptable.
8. I wouldn't change my sexual orientation, even if I could.

All items except 1 and 3 were recoded, and responses were averaged across all eight items. Participants who skipped more than two items were not included.

Participants were asked whether they had experienced any sort of violence due to their sexual orientation in the past year. Responses were categorized as: none, symbolic violence (being mobbed, insulted, or mocked), or physical violence.

HIV-related stigma was included in our analysis as two separate variables. The first, assessing participants views of HIV-infected individuals, was based on the extent to which participants agreed with the following six items:

1. I would not kiss an HIV-positive person on the mouth.
2. I do not want to have any personal contact with HIV-positive people.
3. I cannot imagine having a steady partnership with an HIV-positive person.
4. It requires a certain amount of stupidity to become infected with HIV these days.

5. Those who are currently infected with the HIV virus deserve it.
6. HIV-positive people are irresponsible.

The second measure asked participants to rate how likely it was that they would experience the following six difficulties if they were to be infected with HIV:

1. Friends and acquaintances would think that I failed.
2. Friends and acquaintances would rebuke me about my HIV infection.
3. My family would be disappointed with me.
4. Potential sex partners would reject me.
5. Family and friends would avoid me or want nothing to do with me.
6. I would experience problems in the workplace or at school.

Responses were then recoded and averaged.

Use of establishments/resources catering to gay men

Participants' use of establishments catering toward gay men was assessed by asking participants how often they had visited the following types of places in the past year:

1. Community center, organization, or other group for gay men
2. Cafes or bars
3. Clubs
4. Darkrooms, sex clubs, or open sex parties
5. Private sex parties
6. Gay saunas or porn theaters
7. Other cruising locations
8. Chat, dating, or other internet sites, including smartphone apps

Participants who reported visiting locations many times a week or month were coded as regular visitors, and participants were then labeled as having: no regular contact with establishments that cater to gay men, regular contact with bars and clubs, regular contact with locations with opportunities for on-site sex, or regular contact with bars and clubs and with locations with opportunities for on-site sex. In addition, participants were asked what proportion of their partners in the past year they had met online or through smartphone apps.

Knowledge of HIV and ART

Participants were asked how often they had actively sought out information on HIV in the past year (sometimes, regularly, or never).

Participants were also asked the following four questions concerning ART and could respond that they knew the information and felt well-informed, knew the information but did not feel well-informed, or had never heard the information before:

1. Did you know that there are medications that can be used to treat HIV infection?
2. Have you heard that HIV medications can lower the levels of HIV virus in the body of an HIV-positive person so much that sexual transmission is almost impossible?
3. Have you ever heard about post-exposure prophylaxis? (plus short description of PEP)
4. Have you ever heard about pre-exposure prophylaxis? (plus short description of PrEP)

Attitudes toward ART

Participants' attitudes toward ART were assessed using four separate variables, derived from participants' agreement with the following eight statements:

1. If I were infected with HIV today, I could live a long and healthy life thanks to HIV medication.
2. Today, HIV is a manageable, chronic infection, similar to diabetes.
3. Since HIV medication became available, I have been less concerned about HIV.
4. If I were infected with HIV today, that would not be so bad.
5. If an HIV-positive person regularly take medication, it is almost impossible to be infected by him.
6. An HIV-positive partner with a low viral load is less likely to infect someone than an HIV-positive partner with a high viral load.
7. I would not be worried about getting HIV if I had sex with an HIV-positive partner whose viral load was very low.
8. If my HIV-positive partner and I forgot to use condoms, but he was on HIV medication, I would not be worried.

Questions 1 and 2 assess confidence in the effectiveness of ART, questions 3 and 4 assess the extent of reduced worry about HIV due to ART, questions 5 and 6 assess participants' optimism concerning ART's effectiveness on HIV transmission, and questions 7 and 8 assess the extent to which worries about contracting HIV are reduced due to ART.

Attitudes toward condoms

Participants' attitudes toward condoms were assessed by asking participants the extent to which they agreed with the following statements:

1. Condoms disrupt sex.
2. If I were to insist on using a condom, my partner would assume I was HIV-positive.
3. If I were to insist on using a condom, my partner would assume I was HIV-negative.

Additionally, participants were asked whether they were currently carrying condoms in their bag or had any at home.

Satisfaction with sex life

Participants rated their satisfaction with their sex life as "very satisfied," "fairly satisfied," "fairly unsatisfied," or "very unsatisfied." We simplified this as "satisfied" vs. "unsatisfied."

Substance use

Alcohol consumption is assessed using the AUDIT-C scale, which takes into account participants' responses to the following three items:

1. How often do you consume an alcoholic beverage?
2. How many drinks do you usually have on a given day?
3. How often do you drink six or more alcoholic beverages in one sitting?

Participants were then asked to indicate how often they used a variety of other substances in the past year. They were categorized as using none, one, or more than one substance. Those reporting drug use several times per week or month were coded as being "regular" users, whereas all other participants reporting use of at least one of the above substances were coded as "occasional" users. We also included a simple use vs. nonuse variable in our analyses.

Willingness to use home tests or PrEP

Participants were provided with a short description of home testing, then asked if they would use this type of test. Similarly, participants were given information on PrEP, and asked if, in principle, they would take PrEP in order to reduce their risk of contracting HIV. For both questions, participants could respond “yes,” “no,” or “need more information.”

Reasons for not testing

Participants self-reported reasons for not testing ever or in the past 5 years. We combined the responses “I am afraid I will have to talk about my sexual behaviors” and “I do not want to be lectured about my sexual behaviors” as “I don’t want to discuss my sexual behaviors.” Additionally, participants who were concerned that others might learn about their attraction to men or assume they were HIV-positive were combined for analyses. Reporting that testing centers were too far away or that testing was too expensive was likewise combined. Finally, various infrequently endorsed options, including “I can always be tested later, since treatment options are so good,” “I refuse to be tested on principle,” “I had a bad experience at my last test,” and “since I tested negative before, I think it is difficult for me to be infected” were added to the “other” category.

2. Sensitivity Analysis

In our original analysis, we include survey respondents regardless of whether they report any unprotected sex with their steady partners in the past year, mostly in order to avoid reducing our sample size further. Because men reporting no UAI with their steady partner are at very low risk of transmitting HIV to their steady partners, regardless of outside risk behaviors and HIV testing, we performed a sensitivity analysis in which we removed all individuals reporting no UAI with their steady partners from our dataset (Table S1).

After excluding participants not reporting UAI with their steady partners, we are left with 1289 respondents (230 in the outcome group, 1059 in the reference group). Bivariate analyses revealed that the odds of a participant belonging to the outcome group were significantly higher among those who: had not discussed relationship type with their steady partner; lived in a city with fewer than 100,000 residents; had not graduated from high school; reported being unaware of their steady partner’s status or that their steady partner had never been tested; had a primary doctor who did not know that the participant was attracted to men; reported low levels of social support; had never sought professional help for psychological symptoms; reported higher internalized homophobia; did not report keeping condoms in their house or bag; agreed that condoms disrupt sex; felt that sexual partners would assume they were HIV-positive, or would not necessarily assume they were HIV-negative, if they insisted on condoms; did not feel well-informed about ART; did not feel well-informed about ART’s ability to reduce HIV infectivity; felt less concerned about HIV in general due to ART; did not seek out information about HIV in the past year; or were not aware of PEP.

In multivariate analyses, participants were more likely to belong to the outcome group if they reported their steady partner’s status to be “unknown” or felt that condoms disrupted sex. Participants’ odds of belonging to the outcome group were reduced if their primary doctor knew they were attracted to men, they believed that partners would assume they were HIV-negative if they insisted on condoms, they had sought out information on HIV in the past year, or they felt well-informed about PEP. Among those who believed that partners would

automatically assume they were HIV-negative if they insisted on condoms, reduced concern about HIV due to ART was associated with an increase in the likelihood of belonging to the outcome group.

We note only minor differences between this model and the model presented in the main text. In bivariate analyses, “outness” among heterosexual friends was found to be significant in our main analysis, whereas here it was not. Here, having ever seen a professional due to psychological symptoms was significant, although this relationship was not seen in the main analysis. Additionally, here individuals reporting no clear opinion on whether condoms disrupt sex were not more likely to belong to the outcome group, despite this associated being observed in our main analysis.

In multivariate analyses, presence of condoms in the participant’s home or bag was not found to significantly improve the model. For this reason, the interaction between this variable and seeking out information about HIV was also not included. As in bivariate analyses, only explicitly agreeing that condoms disrupted sex increased the likelihood of belonging to the outcome group. Finally, “regularly” and “occasionally” seeking out information on HIV were found to impact the outcome differently, whereas in our main model the levels could be combined without damaging model fit.

Because neither of the variables found to disagree in bivariate analyses were included in either multivariate model, and because the final multivariate models differed only very slightly, we conclude that our inclusion of individuals reporting no UAI with their steady partners did not impact our results.

**Table S1. Results of bivariate and multivariate sensitivity analyses**. Variables in bold remain significant in the multivariate model.		
	Unadjusted OR (95% CI)	Adjusted OR (95% CI)
Relationship type		
Monogamous	1.00 (ref)	1.00 (ref)
Open	0.73 (0.52-1.04)	0.72 (0.46-1.14)
Undecided	1.58 (1.02-2.44)	1.14 (0.64-2.01)
City of residence >100,000 people	0.67 (0.50-0.89)	
High school diploma	0.69 (0.51-0.92)	
**Steady partner HIV status**		
**Negative**	**1.00 (ref)**	**1.00 (ref)**
Never tested	2.51 (1.67-3.74)	1.57 (0.90-2.68)
**Unknown**	**4.00 (2.66-5.98)**	**2.83 (1.64-4.81)**
**Doctor knows participant is attracted to men**	**0.48 (0.36-0.65)**	**0.49 (0.33-0.72)**
Social support		
High	0.50 (0.31-0.84)	
Medium	0.75 (0.47-1.23)	
Low	1.00 (ref)	
Ever sought professional help for psychological symptoms	0.71 (0.52-0.96)	
Internalized homophobia	1.15 (1.01-1.31)	
Condoms in house or bag	0.35 (0.22-0.54)	
**Feels that condoms disrupt sex**		
**Strongly agrees**	**3.95 (2.64-5.96)**	**3.11 (1.86-5.22)**
**Partially agrees**	**1.57 (1.08-2.29)**	**1.65 (1.06-2.61)**
**Does not agree**	**1.00 (ref)**	**1.00 (ref)**
Difficult to answer	1.67 (0.76-3.39)	2.07 (0.83-4.77)
Feels that partner will assume he is HIV-positive if he insists on condoms	2.50 (1.65-3.74)	
**Feels that partner will assume he is HIV-negative if he insists on condoms**	**0.66 (0.48-0.89)**	**0.23 (0.09-0.59)**
Has ever heard of ART		
Yes, feels well-informed	0.32 (0.16-0.68)	
Yes, but does not feel well-informed	0.63 (0.32-1.32)	
No	1.00 (ref)	
Has heard that ART can reduce infectivity		
Yes, feels well-informed	0.58 (0.39-0.85)	
Yes, but does not feel well-informed	0.77 (0.54-1.12)	
No	1.00 (ref)	
Reduced concerns about HIV due to ART availability	1.64 (1.30-2.06)	1.39 (0.96-1.99)
**Sought information on HIV in past year**		
**Regularly**	**0.28 (0.14-0.51)**	**0.40 (0.17-0.84)**
**Occasionally**	**0.45 (0.32-0.64)**	**0.57 (0.38-0.86)**
**Never**	**1.00 (ref)**	**1.00 (ref)**
**Has ever heard of PEP**		
**Yes, feels well-informed**	**0.38 (0.24-0.59)**	**0.46 (0.26-0.79)**
Yes, does not feel well-informed	0.60 (0.43-0.85)	0.67 (0.44-1.01)

**No**	**1.00 (ref)**	**1.00 (ref)**
**Feels partner will assume he is HIV-negative if he insists on condoms * Reduced concern about HIV due to ART availability (interaction)**	**NA**	**2.01 (1.16-3.50)**
